# Validated High-Performance Liquid Chromatographic Method for the Estimation of Rosuvastatin Calcium in Bulk and Pharmaceutical Formulations

**Published:** 2011-12

**Authors:** Safwan Ashour, Soulafa Omar

**Affiliations:** *Analytical Biochemistry Laboratory, Department of Chemistry, Faculty of Science, University of Aleppo, Aleppo, Syria*

**Keywords:** rosuvastatin calcium, liquid chromatography, pharmaceutical dosage form

## Abstract

A reversed-phase high-performance liquid chromatographic method was developed and validated for the determination of rosuvastatin calcium in pharmaceutical dosage forms. The determination was performed on a Nucleodur column C_8_ (250 × 4.6 mm i.d., 5 μm particle size); the mobile phase consisted of a mixture of 0.1M formic acid and methanol (25:75, v/v), pumped at a flow rate 1.0 mL min^-1^. The photodiode array detector was operated at 280 nm. The retention times for rosuvastatin and fluvastatin, which was used as internal standard, were 3.98 and 7.78 min, respectively. Linearity range (*r*^2^ better than 0.999, *n*=6) was 3.0-1602.0 μg mL^-1^ with limit of detection value of 0.12 μg mL^-1^. The precision of the method was demonstrated using intra- and inter-day assay RSD% values which were less than 2.40%, while the recovery was 99.86-102.86%. The method was applied in the quality control of commercial tablets and content uniformity test and proved to be suitable for rapid and reliable quality control.

## INTRODUCTION

Rosuvastatin calcium is chemically bis [(E)-7 [4-(4-fluorophenyl)-6 isopropyl- 2-[methyl (methyl-sulphonyl) amino] pyrimidin-5-yl] (3R, 5S) -3,5-dihydroxyhept-6-enoic acid] Calcium salt. It is a lipid-lowering drug (1, 2). It inhibits the enzyme 3-hydroxy-3-methyl glutaryl coenzyme A (HMG-CoA) reductase, the rate limiting enzyme that converts HMGCoA to mevalonate a precursor of cholesterol and thereby checks the synthesis of cholesterol. Rosuvastatin is used in the treatment of hyper-cholesterolemia and dyslipidemia (3, 4). To the best of our knowledge, there is no official (pharmacopoeial) method has been found for the assay of rosuvastatin in its formulations. However, HPTLC (5, 6), one RP-HPLC (7) and spectrophotometric (8-10) methods have been reported for the quantification of rosuvastatin calcium in pure drug and in pharmaceutical formulations. Several methods have also been reported for the determination of rosuvastatin in biological fluids. These include HPLC with ultraviolet detector (11), few solid phase extraction using tandem MS (12, 13) and few liquid chromatography/tandem mass spectrometry (LC/MS/MS) (14-18).

The objective of this work was to develop and validate a new quantitative reversed- phase high performance liquid chromatographic method, coupled with photodiode array detector and uses a simple mobile phase composition, as an alternative technique for quality control of rosuvastatin calcium products.

## EXPERIMENTAL

### Chromatographic system

Chromatographic analysis was performed on a modular HPLC system, Hitachi (Japan) consisted of binary pump (L-2130, flow rate range of 0.000-9.999 mL min^-1^), auto sampler (L-2200, injection volume of 0.1-100 μL), column oven (L-2350, temperature range of 1-85°C) and ultraviolet detector (L-2455, 190-850 nm) operated at wavelength of 280 nm and a quartz flow cell (10 mm path and 17 μL volume). Separation was achieved on a reversed phase Nucleodur C_8_ column (250 × 4.6 mm, 5 μm particle size, Macherey-Nagel Germany). The mobile phase was a mixture of a 0.1M formic acid and methanol (25:75, v/v) and was filtered and degassed by ultrasonic agitation before use. The mobile phase was prepared weekly and was delivered at a flow rate of 1.0 mL min^-1^. Data were monitored and processed using automation system software. Peak areas were integrated automatically by computer using the Ezchrom Elite Hitachi software program. The injection volume was 10 μL. The system was operated at ambient temperature.

### Chemicals

HPLC grade methanol and water were purchased from Merck (Darmstadt, Germany). Analytical reagent grade formic acid from Surechem Products LTD (England) was used to prepare the mobile phase.

### Materials

**Figure 1:**
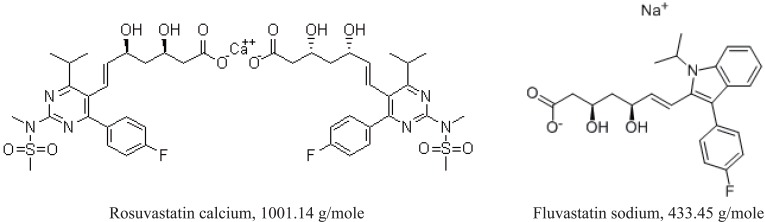
The chemical structure of rosuvastatin calcium and fluvastatin sodium (I.S.).

Rosuvastatin calcium (RSVS) was supplied by MSN Laboratories Ltd, India [(C_22_H_27_FN_3_O_6_S)_2_•Ca = 1001.14 g/mole], and its purity was found to be 99.70%. The internal standard (Fluvastatin sodium, FVS, C_24_H_26_FNO_4_•Na = 433.45 g/mole) was obtained from Zhejiang Materials Industry Chemical Group Co. LTD, China. The chemical structure of RSVS and FVS is given in Figure 1. Tablets containing rosuvastatin calcium: Rosuvastatin 5, 10 and 20 mg (Balsam pharma Co., Syria) and Crestomed 10 and 20 mg (Biomed Pharma Co., Syria).

### Standard solutions

Standard solution of RSVS was prepared by direct weighing of standard substance with subsequent dissolution in methanol. The concentration of the stock standard solution was 2.0 mg mL^-1^. Stock standard solution of FVS 1.0 mg mL^-1^ was prepared by dissolving appropriate amount of the compound in methanol. These solutions were stored in the dark at 2-8°C and were found to be stable for ten days at least. A series working standard solutions of RSVS (3.0-1602.0 μg mL^-1^) were prepared by diluting the stock standard solution with the methanol. In each sample 1mL of FVS was added. Standard solutions were found to be stable during the analysis time.

### Calibration curve

To construct the calibration curve five replicates (10 μL) of each standard solution were injected immediately after preparation into the column and the peak area of the chromatograms were measured. Then, the mean peak area ratio of RSVS to that of the internal standard was plotted against the corresponding concentration of RSVS (3.0-1602.0 μg mL^-1^) to obtain the calibration graph (Table 1).

**Table 1:** Calibration data for the estimation of rosuvastatin by HPLC

Parameters	Rosuvastatin

Optimum concentration range (μg mL^-1^)	3.0-1602.0
Regression equation for the peak area of RVS vs. concentration of RVS in μg/mL, *A*_RSVS_ = 0.345*C*_RSVS_ + 3.367	
Correlation coefficient (r^2^)	0.9999
Standard deviation of slope	0.0014
Standard deviation of intercept	0.0135
Regression equation for the ratio of peak area of RSVS to that of I.S. (FVS) vs. concentration of RSVS in μg/mL, *R*_RSVS/FVS_ = 0.006*C*_RSVS_ + 0.062	
Correlation coefficient (r^2^)	0.9999
Standard deviation of slope	0.0002
Standard deviation of intercept	0.0011
Limit of quantification, LOQ (μg mL^-1^)	0.39
Limit of detection, LOD (μg mL^-1^)	0.12

### Assay procedure for dosage forms

Twenty tablets containing RSVS were weighed and finely powdered. Five accurately weighed quantities of the powder equivalent to 50 mg of RSVS were transferred into 25 mL separated volumetric flasks. A 20 mL of methanol was then added to each flask and the mixture was sonicated for 10 min. Then, the volume of each mixture was adjusted to 25 mL with methanol. The sample solutions were filtered and a suitable concentration was prepared in volumetric flasks containing 1 mL of the internal standard FVS. Finally, 10 μL of each diluted sample was injected into the column. Peak area ratios of RSVS to that of FVS were then measured for the determination. RSVS concentrations in the samples were then calculated using peak data and standard curves.

### Optimization procedure

On the basis of the optimization procedure the following factors were selected and tested in the experimental design: (A) volume percent of methanol (65-90%), (B) flow rate range (0.7-1.4 mL min^-1^) and (C) concentration of formic acid (0.05-0.7 M). Factor levels are given in parenthesis. Experimental design indicates that the best conditions for separation of RSVS from internal standard (FVS) are at mobile phase composition: formic acid solution (0.1M) and methanol (25:75, v/v).

### Validation

The standard curve was a plot of the peak area ratios of RSVS-FVS versus the corresponding concentrations of RVS in the standard curve samples. The linearity of the standard curve was evaluated using least-squares linear regression analysis. To determine recovery of RSVS at concentrations of 3, 20, 50, 200, 400, 620, 1200 and 1602 μg mL^-1^ and of FVS at the concentration used in the assay (100 μg mL^-1^) from bulk or formulations, an identical set of standards prepared in the methanol was analysed. Absolute recoveries at each concentration were measured by comparing the response of pre-treated standards with the response of standards which had not been subjected to sample pre-treatment. Intra- and inter-day coefficients of in variation of the assay were determined by the analysis of five samples at each concentration on the same day and of six samples at each concentration on 4 different days, respectively. The limit of quantification for this assay is defined as the lowest concentration of RSVS that can be detected.

## RESULTS AND DISCUSSION

### Chromatography and selectivity

**Figure 2:**
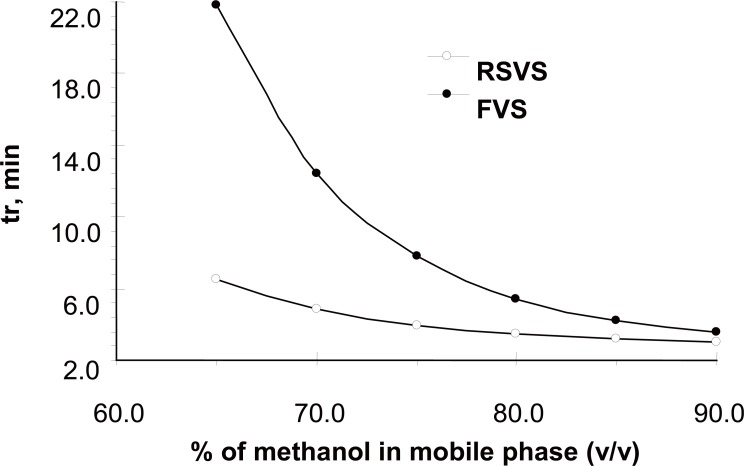
Plots of the retention time *vs.* methanol concentration in the mobile phase of RSVS and FVS.

**Figure 3:**
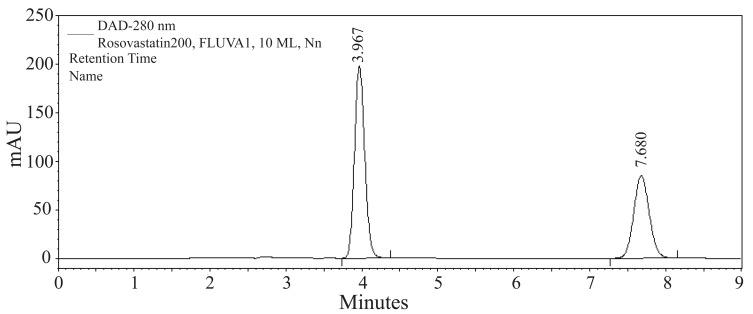
A typical chromatogram of a mixture of RSVS (200 μg mL^-1^) and FVS (100 μg mL^-1^) at retention times 3.98 and 7.78 min, respectively. Chromatographic conditions: RP-HPLC on C_8_ column; mobile phase: 0.1 M formic acid: methanol (25:75, v/v); flow rate 1.0 mL min^-1^ and detection at 280 -nm.

The goal of this study was to develop HPLC assay for the analysis of RSVS drug in pharmaceutical dosage form. Initial studies to develop HPLC assay involved the use of C_18_ and C_8_ columns with various mobile phases containing acetonitrile- or methanol-aqueous formic acid buffers. The C_8_ column was chosen for further studies since it produced sharp and symmetrical peaks. The final selective HPLC mobile phase consisting of MeOH-HCOOH. The effect of composition of the mobile phase on the retention time of RSVS and the internal standard, FVS, was investigated. Results of the effect of methanol in the mobile phase are presented in Figure 2. An increase in the percentage of methanol decreases the retention of compounds; RSVS and FVS. Increasing methanol concentration to more than 80% RSVS peak is eluted with the solvent front, while at methanol concentration lower than 70% the elution of FVS peak is seriously delayed. The optimum methanol concentration was found to be 75%. The effect of pH in the chromatographic elution of both compounds was also investigated by changes the concentration values of the aqueous component of the mobile phase from 0.05 to 1.0 M. For all experimental concentration values, the drugs are eluted in order of RSVS and FVS. A concentration value of 0.1 M HCOOH was chosen for the optimum separation of the compounds, as at this concentration the analyte peaks were well defined and resolved. The optimum wavelength for detection was at 280 nm, at which the best detector responses for all substances were obtained. The specificity of the HPLC method is illustrated in Figure 3 where complete separation of the compounds was observed. RSVS was eluted at 3.98 min, while the internal standard FVS was eluted at 7.78 min.

### Linearity and limits of quantification and detection

Standard curve of RSVS was linear (r^2^>0.99) over a wide range of the concentrations 3.0-1602.0 μg mL^-1^, in relation with similar study (7). Straight line for RSVS was obtained, when the area of the peaks were plotted versus concentration (Table 1). Also, Linear relationship was obtained between the peak area ratio of RSVS to that of the internal standard FVS and the corresponding concentration of RSVS (3.0-1602.0 μg mL^-1^), as shown by the equations presented in Table 1.

The minimum level at which the investigated compound can be reliably quantified (limit of quantification, LOQ) and detected (limit of detection, LOD) were determined experimentally. LOD was expressed as the concentration of drug that generated a response to three times of the signal to-noise (S/N) ratio, and LOQ was 10 times of the S/N ratio. The LOD of RVS attained as defined by IUPAC (19), LOD_(*k*=3)_=*k × S_a_/b* (where *b* is the slope of the calibration curve and S_a_ is the standard deviation of the intercept), was found to be 0.12 μg mL^-1^. The LOQ was also attained according to the IUPAC definition, LOQ_(*k*=10)_=*k × S_a_/b*, and was found to be 0.39 μg mL^-1^. So, the proposed method is more sensitive than the similar method (7).

### Accuracy and precision

The precision and accuracy of the method were evaluated by intra- (analysis of standard solutions of RSVS in replicates of five in the same day) and inter-day (analysis of standard solutions of RSVS in replicates of five on 4 different days from day 1 to 10 after preparation) assay variance (Table 2). The standard deviation, relative standard deviation, recovery and relative percentage error of different amounts tested were determined, as recorded in Table 2. The accuracy of the method is indicated by the excellent recovery (99.86-102.86%) and the precision is supported by the low standard deviation. Table 2 shows that the percent error of the method was always less than 2.86%; therefore, it was concluded that the procedure gives acceptable accuracy and precision for the analyte.

**Table 2:** Accuracy and precision of within and between run analysis for the determination of rosuvastatin by HPLC

Nominal concentration (μg.mL^-1^)	Assayed concentration (μg mL^-1^)			
	Mean ± SD	RSD (%)	Recovery (%)	Relative error (%)

Intra-day (n=6)				
3.00	3.08 ± 0.06	2.17	102.86	2.86
20.00	19.97 ± 0.30	1.53	99.86	-0.14
50.00	50.47 ± 0.49	0.97	100.94	0.94
200.00	202.14 ± 1.87	0.92	101.07	1.07
400.00	401.70 ± 3.04	0.75	100.42	0.42
620.00	622.43 ± 3.27	0.52	100.39	0.39
1200.00	1205.95 ± 3.77	0.31	100.49	0.49
1602.00	1603.87 ± 3.97	0.25	100.11	0.11
Inter-day (n=6)				
3.00	3.07 ± 0.07	2.40	102.50	2.50
20.00	20.16 ± 0.30	1.48	100.80	0.80
50.00	50.26 ± 0.50	0.99	100.53	0.53
200.00	201.35 ± 1.47	0.73	100.67	0.67
400.00	400.31 ± 2.52	0.63	100.08	0.08
620.00	619.57 ± 2.74	0.44	99.93	-0.07
1200.00	1202.65 ± 4.48	0.37	100.22	0.22
1602.00	1600.83 ± 3.52	0.22	99.92	-0.08

### Application of the assay

**Figure 4:**
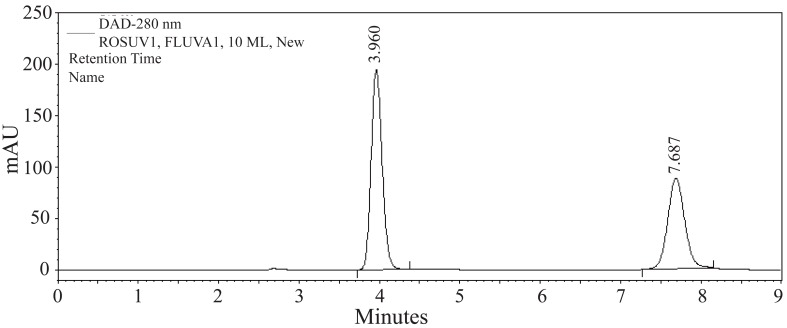
A typical chromatogram of a mixture of RSVS (200 μg mL^-1^) and the internal standard, FVS (100 μg mL^-1^) in methanol, prepared from Rosuvastatin 20mg tablets. Chromatographic conditions: C_8_ column; mobile phase: 0.1 M formic acid and methanol (25:75, *v/v*); flow rate 1.0 mL min^-1^ and detection at 280 nm.

The present method has been successfully applied for the determination of rosuvastatin calcium in five different rosuvastatin preparations. The resulted chromatogram has been shown in Figure 4. Student’s t-test was used for statistical analysis of the data and statistical significance was defined at the level of *P*<0.05. The results obtained with the proposed method were compared with the official method (8) and have been shown in Table 3. Good agreement with results obtained by the official method was observed. The proposed method is simple, rapid, accurate, highly sensitive and suitable for the routine quality control without interference from the excipients and additives such as starch, glucose and lactose.

**Table 3:** Determination of RSVS in pharmaceutical formulations by the proposed method and official method

Product^a^	Pharmaceutical company (country of origin)	% Found^b^ ± SD	
		Proposed method	Official method (8)

Rosuvastatin 5 mg	Balsam pharma Co. (Syria)	104.01 ± 0.15	103.35 ± 0.11
		*t*=2.02	*t*=2.12
		*F*=1.86	
Rosuvastatin 10 mg	Balsam pharma Co. (Syria)	103.50 ± 0.45	103.00 ± 0.34
		*t* =1.16	*t* =1.34
		*F*=1.75	
Rosuvastatin 20 mg	Balsam pharma Co. (Syria)	100.09 ± 0.33	100.06 ± 0.26
		*t*=1.94	*t*=2.19
		*F*=1.61	
Crestomed 10 mg	Biomed Pharma Co. (Syria)	101.50 ± 0.41	101.80 ± 0.34
		*t*=2.10	*t*=2.21
		*F*=1.45	
Crestomed 20 mg	Biomed Pharma Co. (Syria)	100.05 ± 0.38	100.39 ± 0.31
		*t*=2.14	*t*=2.28
		*F*=1.50	

a The dose is 5, 10 and 20 mg expressed as rosuvastatin calcium for all products;

b Five independent analyses. At 95% confidence level t-value is 2.776 and F-value is 6.26.

## CONCLUSION

A convenient, accurate, precise and inexpensive RP-HPLC method has been developed for the routine analysis of rosuvastatin calcium in bulk and in tablet dosage form. The method uses a simple mobile phase composition, which can be easily prepared, along with the short analytical run time of 9 min for the drug and internal standard. Hence, this method can be used for the analysis of large number of samples. The method provides a linear response across a wide range of concentrations. The sample recoveries from all formulations were in good agreement with their respective label claims, which suggested non-interference of formulations excipients in the estimation. Moreover, the present method is fast with respect to analysis time as compared to sophisticated chromatographic techniques. The method provided excellent specificity and linearity with a limit of quantification of 0.39 μg mL^-1^ and limit of detection of 0.12 μg mL^-1^. The major advantages of this method are the wide range of linearity and the high sensitivity.
